# Association between intraoperative hypotension during brain tumor resection and postoperative delirium: A secondary analysis of a randomized controlled trial

**DOI:** 10.1371/journal.pone.0334094

**Published:** 2025-10-29

**Authors:** Yishuang Wu, Yue Ren, Shu Li, Min Zeng, Jie Wang, Muhan Li, Yuming Peng

**Affiliations:** 1 Department of Anesthesiology, Beijing Tiantan Hospital, Capital Medical University, Beijing, PR China; 2 Department of Outcomes Research Consortium, Cleveland, Ohio, United States of America; University of Split Faculty of Medicine: Sveuciliste u Splitu Medicinski fakultet, CROATIA

## Abstract

**Objective:**

Postoperative delirium is a common complication after neurosurgery. The association between intraoperative hypotension and postoperative delirium in the neurosurgical population is unknown.

**Design:**

This is a secondary analysis of a randomized controlled study

**Setting:**

Adults scheduled for elective craniotomy under general anesthesia were included in 1 study center.

**Participants:**

Of 260 patients, a total of 240 participants are included for final analysis after excluding patients without intraoperative blood pressure data.

**The primary outcome measures:**

The primary outcome was the occurrence of delirium within the first 5 postoperative days, assessed with the Confusion Assessment Method or a 3-minute Diagnostic interview for the Confusion Assessment Method.

**Results:**

A total of 240 patients were included (median age, 45 years), and 83(35%) patients experienced postoperative delirium. Curves of lowest mean arterial pressure versus stroke incidence suggested a threshold at 65 mmHg. There was no association between duration below 65 mm Hg and postoperative delirium (odds ratio, 1.01; 95% confidence interval, 0.96, 1.06). The odds ratio for duration below 65 mm Hg for 10 minutes was 1.03 (95% confidence interval, 0.97, 1.09) adjusted by history of hypertension, age > 45 yr, tumor volume, tumor type of glioma, preoperative Mini-Mental State Examination (MMSE) >26, and dexmedetomidine infusion which were all indicated the independent risk factors for delirium.

**Conclusions:**

The current results could not indicate intraoperative hypotension of mean arterial pressure lower than 65 mmHg associated with delirium after frontotemporal brain tumor resection.

**Trial registration:**

ClinicalTrials.gov NCT04674241

## Introduction

Postoperative delirium (POD) is a cognitive disorder with acute and fluctuating impairment of attention, consciousness, and cognition after surgery, with peak incidence occurring on postoperative 1–5 days [1–3]. The occurrence of POD is associated with a prolonged hospital stay, higher medical expenses, long-term cognitive dysfunction, and increased mortality [4]. Neurosurgical populations are more likely to develop POD, with a reported incidence ranging from 10% to 46% [4–9].

Intraoperative hypotension was reported to contribute to POD [10,11]. It may be related to the hypoxia of perioperative cerebral ischemia, which leads to inadequate blood supply to the brain and further causes hippocampal cell damage and mitochondrial structural dysfunction [12]. Patients with low blood pressure below the individual limit of cerebral pressure autoregulation may be at risk of hypoperfusion and cause POD [13,14]. On the other hand, some studies suggested that neither intraoperative nor postoperative hypotension was associated with POD [15,16]. However, in the previous studies, hypotension was analyzed dichotomously, and the definition of hypotension was only based on clinical experience. Moreover, whether the duration of a specific hypotension threshold is related to POD remains unknown.

Therefore, we aimed to assess the association between hypotension and POD in patients who received supratentorial brain tumor resection in the previous randomized controlled trial. We tested the primary hypothesis that the duration, the level, or the area under the curve of intraoperative hypotension was associated with POD.

## Methods

This was a secondary analysis of a randomized, placebo-controlled trial registered at www.ClinicalTrials.gov on December 19, 2020 (NCT04674241, Principal Investigator: Yuming Peng). The trial protocol was approved by the Chinese Ethics Committee of Registering Clinical Trials (ChiECRCT-20200436) on November 10, 2020, and has been published [16]. Patients were enrolled from January 2021 to December 2021. The anesthesia management had been described in detail [9,16]. After the patient was admitted to the operating room, routine monitoring should be conducted. Heart rate and mean arterial pressure (MAP) were controlled within ± 20% of baseline values. The original trial was a parallel-group, 1:1 randomized trial investigating the effect of dexmedetomidine on supratentorial tumor resection in relation to POD. Patients were randomized on the morning of surgery by an independent investigator who was not otherwise involved in the trial in a 1:1 ratio, stratified by center, based on computer-generated codes in blocks of four patients. At 10 minutes after induction of anesthesia and tracheal intubation, patients were randomly assigned into the dexmedetomidine group and were given a loading dose of dexmedetomidine 0.6 mg kg-1during 10 minutes, and then continuous infusion at a rate of 0.4 mg kg^-1^h^-1^ until the start of dural closure. Subjects in the placebo group were given comparable volumes of normal saline. The primary endpoint was the incidence of delirium during the ﬁrst 5 postoperative days, assessed by the Confusion Assessment Method for Intensive Care Unit (CAM-ICU) for patients in the intensive care unit, or the 3-minute Diagnostic interview for Confusion Assessment Method (3D-CAM) in the ward.

### Participants

All patients were 18 years of age or older with frontotemporal brain tumors scheduled for elective craniotomy under general anesthesia at Beijing Tiantan Hospital. Exclusion criteria were as follows: preoperative Mini-Mental State Examination score <20, allergy to dexmedetomidine, use of psychotropic medication within 30 days before surgery, pregnancy or lactation, with a history of traumatic brain injury, severe bradycardia (heart rate <40 beats min^-1^), with sick sinus syndrome or second-to-third degree atrioventricular block, or severe hepatic or renal dysfunction. We also excluded patients without continuous intraoperative blood pressure recording.

### Intraoperative blood pressure acquisition and processing

Intraoperative blood pressures were extracted from the Anesthesia Information Management System (AIMS, version 5.0, Wangfeng Mingyue Ltd, China). Among the included 240 patients, 237 (99%) patients had invasive blood pressure monitoring using arterial catheters placed in the dorsal pedis or radial arteries. The transducers were placed at the level of the right atrium. The other three patients were only under non-invasive pressure monitoring.

Invasive pressures were recorded at 10-second intervals. The following MAP data were marked as artifacts and removed from the analysis using a user-written Python program algorithm: 1) Invasive blood pressure data during the first 5 minutes corresponding to transducer flush, leveling and zeroing; 2) out-of-range invasive blood pressure, defined as a systolic blood pressure ≥ 300 or ≤ 20 mmHg; 3) sudden changes in systolic blood pressure (≥ 80 mmHg within 1 minute in either direction) or diastolic blood pressure (≥ 40 mmHg within 1 minute in either direction) without any annotation to identify clinical causes; 4) invasive data that remained unchanged for more than 5 minutes, probably from blocked monitoring lines. Pressures were linearly interpolated between measurements during minutes when no blood pressure was recorded or when a value was marked as an artifact. Detailed methods of intraoperative blood pressure cleaning were described in previous studies [17,18].

### Assessment and outcomes

The primary outcome was the incidence of POD in the first 5 days after surgery. POD was assessed twice daily (between 8–10 am and 6–8 pm) in combination with the Richmond Agitation Sedation Scale (RASS) [19]; if patients had a RASS sedation score greater than −4, POD was assessed using the Confusion Assessment Method for Intensive Care Unit (CAM-ICU) for ICU patients [20], and the 3-minute Diagnostic interview for Confusion Assessment Method (3D-CAM) for ward assessments [21]. These screening tools for delirium were widely used after neurosurgery [22–25]. All diagnostic tests were performed by investigators blinded to the intraoperative dexmedetomidine infusion. The investigators were specially trained by a neurologist.

### Statistical analysis

We included 240 patients who met study criteria. By post-hoc analysis, we had 80% power to detect an odds ratio of 1.45.

Patients are classified according to whether they developed POD within 5 days, and baseline characteristics and variables related to surgery and anesthesia are compared between groups. Categorical data are reported as counts (percentages) and analyzed using 2-tailed chi-squared tests with continuity correction or the Fisher exact test. Continuous variables are presented medians and interquartile ranges (IQR) and are compared with students’ t-tests when normally distributed or otherwise with Mann-Whitney tests. Variables with an absolute standardized difference $>\text{0}.\text{266}(\text{1}.\text{96}*\sqrt{}(\frac{\text{1}}{\text{8}\text{3}}+\frac{\text{1}}{\text{1}\text{5}\text{7}}))$ baseline are identified as imbalanced potential confounders. These variables are adjusted for these characteristics in our multivariable modeling. In addition, because our previous study found that intraoperative infusion of dexmedetomidine can effectively reduce the incidence of POD [9], dexmedetomidine infusion is preset as a variable for adjustment.

To investigate the association between intraoperative hypotension and the incidence of POD, We first identified change points indicative of a harm threshold using logistic regression, with the method reported previously [18,26,27], Data de-noising was performed using the simple moving average method with a width of 5 min to determine the lowest blood pressure for each patient. We then calculated the total time with the lowest MAP and graphed the predicted probability of POD. We performed logistic regression to predict the probability of POD over the range of MAP from 20 mmHg to 100 mmHg. The potential threshold (inflection point) was determined by identifying the second derivative of the regression using the “Kneedle” method in Python [28]. By applying the Kneedle method (developed computationally), we investigated the blood pressure threshold at which the risk of POD changes. A flatter risk curve indicates a decrease in curvature, signaling the emergence of an inflection point. The cumulative duration of hypotension was measured in minutes, as the duration below the harm threshold started when the MAP was below a specific threshold and ended when the MAP exceeded the specific threshold again. The cumulative sum of the areas calculated using the trapezoidal rule was defined as the area under the harm threshold and was expressed in units of mmHg times minutes. The time-weighted average (TWA) MAP under the threshold was derived by dividing the area below the threshold by the duration of anesthesia.

We modeled the incidence of POD using logistic regression, with duration below 65 mmHg as an independent variable. We selected imbalanced variables between groups as confounders, conducted a multicollinearity test on these variables and excluded those with multicollinearity. These variables are adjusted for these characteristics in our multivariable modeling. Results are presented as odds ratios (OR) or adjusted odds ratios (aOR) and their 95% confidence intervals (CI). The Hosmer-Lemeshow goodness-of-fit test and the area under the receiver operating characteristic curve were presented as model diagnostics. All statistical analyses were carried out using Stata/SE 16.0 (StataCorp, TX, USA), and statistical significance was considered at P < 0.05.

## Result

**Fig 1:**
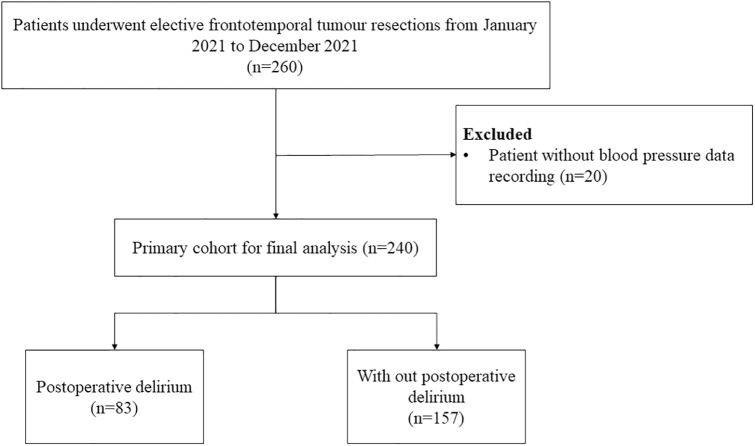
Study flowchart. This is the flowchart of study population.

After excluding patients without intraoperative blood pressure data, a total of 240 patients are included for final analysis (Fig 1). Among the 240 patients, 237 (99%) had invasive blood pressure monitoring, while the remaining 3 (1%) had non-invasive blood pressure monitoring. Eighty-three patients (35%) experienced POD within five days after surgery.

Patients’ baseline characteristics and preoperative neurosurgical features are summarized in Table 1. Age, preoperative hypertension, tumor type, tumor equivalent diameter, Charlson Comorbidity Index, and Mini-Mental State Examination had adjusted standardized differences exceeding our threshold of 0.266. We thus adjusted for these characteristics in our multivariable modeling. The intraoperative and postoperative characteristics are reported in Table 2. Patients who experienced POD received slightly higher doses of propofol and higher volume infusion. The number of patients with intraoperative mannitol infusion and dexmedetomidine infusion was higher in patients who did not experience POD. Furthermore, the median surgery duration was longer in patients who developed POD than in patients who did not (260 minutes vs. 220 minutes, P = 0.001).

**Table 1:** Demographic and clinical characteristics at baseline.

Characteristics	All patients (n = 240)	POD (n = 83)	Without POD (n = 157)	ASD
Age, median (IQR), year	45 (35-53)	48 (38-57)	42 (34-52)	0.456
Sex, female, no. (%)	115 (47.9)	42 (50.6)	73 (46.5)	0.045
Body mass index, median (IQR) ^a^	24.5 (22.4-27.0)	25.0 (22.8-27.1)	24.4 (22.3-26.8)	0.084
**Medical history, No. (%)**				
Smoking history	34 (14.2)	11 (13.3)	23 (14.7)	0.04
Drinking history	25 (10.4)	16(10.2)	9 (10.8)	0.021
Hypertension	30 (12.5)	17(20.5)	13 (8.3)	0.353
Diabetes	12 (5.0)	6 (7.2)	6 (3.8)	0.15
Hypothyroidism	5 (2.1)	2 (2.4)	3 (1.9)	0.034
Stroke	4 (1.7)	3 (3.6)	1 (0.6)	0.208
Allergic history	25 (10.4)	11 (13.3)	14 (8.9)	0.138
Surgery	30 (12.5)	13 (15.7)	17 (10.8)	0.143
**Neurological symptom, No. (%)**				
Headache	53 (22.1)	17 (20.5)	36 (22.9)	0.059
Dizziness	45 (18.8)	12 (14.5)	33 (21.0)	0.172
Vomiting	12 (5.0)	5 (6.0)	7 (4.5)	0.070
Nausea	15 (6.3)	6 (7.2)	9 (5.7)	0.061
Epilepsy	88 (36.7)	33 (39.8)	55 (35.0)	0.098
Cranial nerve deficit	6 (2.5)	2 (2.4)	4 (2.6)	0.009
**Tumor characteristics**				
Tumor type, No. (%)				0.586
Meningioma	47 (19.6)	6 (7.2)	41 (26.1)	
Glioma	181 (75.4)	75 (90.4)	106 (67.5)	
Others ^b^	12 (5.0)	2 (2.4)	10 (6.4)	
Tumor main location, No. (%)				0.194
Temporal	59 (24.6)	25 (30.1)	34 (21.7)	
Frontal	181 (75.4)	58 (69.9)	123 (78.3)	
Side of lesions, No. (%)				0.290
Left	125 (52.1)	51 (61.5)	74 (47.1)	
Right	115 (47.9)	32 (38.6)	83 (52.8)	
WHO classification, No. (%)				0.202
Grade 1–2	127 (52.9)	42 (50.6)	85 (54.1)	
Grade 3–4	102 (42.5)	39 (47.0)	63 (40.1)	
Others ^b^	11 (4.6)	2 (2.4)	9 (5.7)	
Tumor equivalent diameter, median (IQR), mm	35 (26-45)	41 (32–48)	33 (24-41)	0.411
Midline shift, median (IQR), mm	0 (0-1)	0 (0-1)	0 (0−0)	0.315
**Preoperative assessment**				
ASA classification, No. (%)				0.006
I-III	237 (98.8)	82 (98.8)	155 (98.7)	
IV	3 (1.3)	1 (1.2)	2 (1.3)	
Charlson Comorbidity Index, median (IQR)	0 (0-1)	0 (0-1)	0 (0-1)	0.382
Mini-Mental State Examination, median (IQR) ^c^	30 (30–30)	30 (30–30)	30 (30–30)	0.502
Mini-Mental State Examination ≤26, No. (%)	10 (4.2)	9 (10.8)	1 (0.6)	0.450
Preoperative delirium, No. (%)	0 (0.0)	0 (0.0)	0 (0.0)	/

ASA, American Society of Anesthesiologists; POD, postoperative delirium; ASD, absolute standard difference; WHO, World Health Organization; IQR, interquartile range.

^a^ Calculated as weight in kilograms divided by height in meters squared.

^b^ Others include cholesteatoma, epidermoid cyst, subependymoma, and metastasis.

^c^ The maximum Mini Mental State Examination, median score is 30 points. Normal values are < 24 for people with less than post-secondary education, < 23 for those with less than secondary education, < 20 for those with less than primary education.

**Table 2:** Intraoperative and postoperative characteristics.

Characteristics	All patients (n = 240)	POD (n = 83)	Without POD (n = 157)	P values
**Intraoperative medications**				
Midazolam, median (IQR), mg	2 (2.0-2.3)	2 (2–2)	2 (2–3)	0.380
Propofol dose, median (IQR), mg	900 (735-1200)	950 (780-1250)	900 (710-1100)	0.043
Sufentanil dose, median (IQR), (μg)	36 (30–45)	40 (35–45)	35 (30–45)	0.566
Vasopressor, No. (%)	31 (12.9)	12 (14.5)	19 (12.1)	0.605
Mannitol, No. (%)	165 (68.8)	64 (77.1)	101 (64.3)	0.042
Glucocorticoid, No. (%)	26 (10.8)	8 (9.6)	18 (11.5)	0.665
Neuromuscular block reversal, No. (%)	18 (7.5)	6 (7.2)	12 (7.6)	0.908
Dexmedetomidine infusion, No. (%)	120 (50.0)	25 (30.1)	95 (60.5)	0.000
**Intraoperative fluid infusion**				
Total infusion, median (IQR), ml	2500 (2000-2775)	2500 (2200-3000)	2500 (2000-2500)	0.002
Urine, median (IQR), ml	1500 (1200-2000)	1500 (1400-2000)	1500 (1200-1900)	0.083
Estimated blood loss, median (IQR), ml	200 (150-200)	200 (150-300)	200 (150-200)	0.271
Allogeneic red blood cell infusion, No. (%)	5 (2.1)	0 (0.0)	5 (3.2)	0.100
Allogeneic plasma infusion, No. (%)	4 (1.7)	0 (0.0)	4 (2.6)	0.143
Baseline MAP, median (IQR), mmHg	95.3 (86.7-102.8)	96.3 (89.7-105.0)	95.3 (85.3-101.7)	0.204
Duration of BIS < 35, median (IQR), min	29.5 (6-76)	34.5 (5-81.5)	29 (7-76)	0.141
**Tumor resection, No. (%)**				0.328
Complete resection	215 (89.6)	71 (85.5)	144 (91.7)	
Gross total resection	21 (8.8)	10 (12.1)	11 (7.0)	
Subtotal resection	4 (1.7)	2 (2.4)	2 (1.3)	
Surgery duration, median (IQR), min	238 (188-290)	260 (210-310)	220 (180-280)	0.001

MAP, mean arterial pressure; BIS, bispectral index; POD, postoperative delirium; IQR, interquartile range.

Based on the second derivative for the cut-point of approaching zero, we adopted of 65 mmHg as the intraoperative hypotension threshold for POD (S1 Fig). The threshold of 65 mm Hg was corresponded to 68% of the baseline pressure in all patients. A total of 135 patients (56%) experienced intraoperative hypotension less than 65 mmHg (51 with POD (38%) vs 84 without POD (62%)), 193 patients (80%) experienced intraoperative hypotension less than 70 mmHg, and only 70 patients (29%) had intraoperative hypotension less than 60 mmHg. The duration, area, and time-weighted MAP did not differ significantly between patients with and without a POD below 65 mm Hg or thresholds of 60 mm Hg and 70 mm Hg (Table 3). We didn’t find significant association between duration below 65 mm Hg and POD (OR, 1.01; 95% CI 0.96, 1.06). The adjusted OR for every 10 minutes below 65 mmHg was 1.03(95% CI 0.98, 1.09) after adjusted for preoperative MMSE >26 (aOR, 0.58; 95% CI: 0.38, 0.86), intraoperative dexmedetomidine infusion (aOR, 0.26; 95% CI: 0.14, 0.52), history of hypertension (aOR, 3.20; 95% CI: 1.20, 8.49), Charlson Comorbidity Index>0 (aOR, 1.12; 95% CI: 0.49, 2.56), age > 45 years (aOR, 2.82; 95% CI: 1.40, 5.72), tumor type of glioma (aOR, 4.72; 95% CI: 1.82, 12.26) and tumor equivalent diameter >40 mm (aOR, 1.92; 95% CI: 1.00, 3.68) (Table 4). In the multivariable model, the area under the receiver operating characteristic curve for 65 mm Hg was 0.81 (95% CI, 0.76, 0.87) and P = 0.308 in the Hosmer-Lemeshow test.

**Table 3:** Hypotension characteristics for various thresholds.

Characteristics	POD (n = 83)	Without POD (n = 157)	P value
Duration (IQR), min			
Below 70 mmHg	49 (10, 134)	43 (4, 141)	0.211
Below 65 mmHg	7 (0, 39)	5 (0, 38)	0.060
Below 60 mmHg	0 (0, 5)	0 (0, 3)	0.090
AUC – MAP (IQR), mmHg * min			
Below 70 mmHg	131 (15, 512)	113 (4, 567)	0.134
Below 65 mmHg	10 (0, 95)	3 (0, 107)	0.052
Below 60 mmHg	0 (0, 9)	0 (0, 4)	0.151
Time weighted average MAP(IQR), mmHg/ min			
Below 70 mmHg	0.4 (0.0, 1.8)	0.6 (0.0, 1.7)	0.132
Below 65 mmHg	0.0 (0.0, 0.3)	0.0 (0.0, 0.3)	0.052
Below 60 mmHg	0.0 (0.0, 0.0)	0.0 (0.0, 0.0)	0.140

AUC, area under curve; MAP, mean arterial pressure; IQR, interquartile range. POD, postoperative delirium.

**Table 4:** Univariate and multivariate analysis of the association between intraoperative hypotension and POD.

Predictor variables	Univariate		Multivariate	
	Odds Ratio	95% CI	Adjusted odds Ratio	95% CI
Duration below 65 mmHg, 10 min	1.01	0.96, 1.06	1.03	0.98, 1.09
Preoperative MMSE >26, 1 unit	0.54	0.39, 0.76	0.58	0.38, 0.86
Intraoperative Dexmedetomidine infusion	0.28	0.16, 0.50	0.26	0.14, 0.52
History of hypertension	2.85	1.31, 6.22	3.20	1.26, 8.94
Charlson Comorbidity Index >0	2.39	1.36,4.18	1.12	0.49,2.56
Age > 45 years	2.15	1.25, 3.70	2.82	1.38, 5.59
Glioma	4.51	2.02, 10.05	4.72	1.76, 13.41
TED > 40mm	1.03	1.01, 1.05	1.92	1.00, 3.68

In the multivariable model, the area under the receiver operating characteristic curve for 65 mm Hg was 0.81 (95% CI, 0.76–0.87) and P = 0.308 in the Hosmer-Lemeshow test. Multicollinearity was assessed using variance inflation factor, and was not found with significance. MMSE, Mini-Mental State Examination; CI, confidence intervals; TED, tumor equivalent diameter. The formula for TED is the tumor length times its width times its height to the square root.

## Discussion

In this study, we didn’t find a association between intraoperative hypotension, defined as MAP < 65 mmHg, and POD in the supratentorial neurosurgical population. Although in the original study, 47% of patients undergoing brain tumor surgery experienced POD within 5 days after surgery [9] indicating the neurosurgical population is at especially high risk for postoperative cognitive dysfunction and delirium. In the multivariate model for POD, the independent risk factors, such as age, preoperative cognitive injury, glioma, and the protective factor, dexmedetomidine infusion, all have a significant association with POD. Whereas intraoperative hypotension, being a relatively weak risk factor for POD, might not have been found to has statistical significance in the current study with a small sample size of only 240 cases. Hypotension was deemed to be one of the contributors [10,11,13,14]. Several hypotension descriptors and threshold definitions have been reported in previous studies [17,18,26,29].

Intraoperative hypotension induced by increased intracranial pressure, perioperative mannitol infusion, intraoperative brain stem reflex (e.g., Trigeminocardiac reflex), and hemorrhage is common in intracranial tumor resections [30]. Subsequent insufficient cerebral blood supply could result in neuronal damage or distress in the hippocampal CA1 subfield [12], which may cause memory impairment and develop into POD. Moreover, Hypotension is also associated with the breakdown of the blood-brain barrier [31], hence associated with a neuroinflammatory response. The accumulation of inflammatory mediators leads to loss of synaptic plasticity, neuro-apoptosis, and impaired neurogenesis, and ultimately leads to delirium [32,33].

Previous studies indicated hypotension as a risk factor for POD [13,14,34,35]. A higher time-weighted MAP < 65 mmHg was associated with POD during ICU stay [11]. A randomized controlled trial found that MAP maintained from baseline to 10% above the baseline had a lower incidence of POD in the older non-neurosurgical population [14]. Moreover, in cardiac surgery, targeting mean arterial pressure to be higher than the individual patient’s lower limit of cerebral autoregulation decreases the incidence of delirium [13]. However, we did not identify any adjusted or unadjusted association between intraoperative hypotension and POD in this secondary analysis. This may be related to the low incidence and shorter duration of hypotension in the well-controlled trial. In both groups, the median duration below 65 mmHg was 7 minutes or less which was not long enough to establish the association between hypotension and POD. Furthermore, the analyzed population was relatively younger with stable intraoperative hemodynamic parameters, with standardized intraoperative blood pressure management [9]. Therefore, fewer participants were exposed to hypotension with a short duration and subsequently veiled the relation between hypotension and POD.

Preoperative hypertension is common in brain tumor patients with increased intracranial pressure, disrupted blood-brain barrier, and impaired cerebral autoregulation [36]. Elevated blood pressure increases cerebral blood flow in the presence of impaired autoregulation, leading to the transudation of fluid into the pericapillary astrocytes and interstitium of the brain [37]. Blood pressure above the upper limit of cerebral autoregulation contributes to increased cerebral blood flow, resulting in excessive cerebral micro embolic load, endothelial damage, and compromised blood-brain barrier. These aberrations expose patients to hypertensive encephalopathy and neuroinflammation, which has been suggested to contribute to delirium susceptibility [38,39]. The mechanisms still require further research to explore.

The impact of glioma on grey matter volume and functional connectivity in specific areas, including the right dorsal lateral prefrontal cortex, suggested that insufficient compensation for injured brain regions involving cognition might be more vulnerable to suffering from POD [40]. Previous studies have reported a large volume of the tumor was also suggested to be associated with postoperative delirium through decreased compensation of functional areas and impairing blood-brain barrier [41]. That we only detect a mild association between tumor volumes with POD is presumably due to the small difference between groups.

The elderly population (age > 65 years), with brain degeneration [42] and increased systemic comorbidities, was reported to be associated with a higher incidence of POD previously [15,35]. We note, though, in our analysis, a younger age of 45 years would also be susceptible to an increased risk of POD. It was broadly accepted that the volume of the brain and its weight decreased with age at a rate of around 5% per decade after age 40 [43]. Our result suggested, presumably, that a mild degeneration of the brain may contribute to the increased risk of POD.

The major limitation of our analysis is that this is a secondary analysis of a trial, and blood pressure management was not randomized (e.g., to routine care vs. hypotension avoidance). Though all data was prospectively collected, some risk factors for POD, including inflammatory factors such as C-reactive protein and interleukin-6 concentrations, were not measured. And as a single-center study, the results might be influenced by the relatively young population and clinical protocol of the institution. The generalizability of the study might be weak and multicenter studies are needed to confirm the broader applicability. On the other hand, this original trial aimed to maintain intraoperative MAP within ±20% of the baseline. Besides, the younger population and the standardized blood pressure management, only a few patients were exposed to intraoperative hypotension with a short duration. The well-controlled blood pressure in the trial setting might not reflect real-world practice. In this study, a uniform hypotension threshold was calculated and set as the exposure factor for the study population. However, the thresholds for cerebral autoregulation may vary among individuals due to differences in their medical history of hypertension and the characteristics of their brain tumors. This secondary analysis did not incorporate individualized thresholds as exposure factors, and the conclusions drawn may therefore be subject to potential bias. Additionally, neurosurgical patients may be unable to complete RASS score assessments or the Confusion Assessment Method due to postoperative coma or aphasia. These combined factors may compromise the assessment of POD and contribute to misclassification bias. However, the outcome assessors were blinded to minimize bias as much as possible.

## Conclusions

In summary, intraoperative hypotension may not be associated with POD in patients with a supratentorial brain tumor resection population. However, an explicit intervention for intraoperative blood pressure management is needed to explore the causal reference between hypotension and POD in this population.

## Supporting information

S1 Fig The potential threshold prediction using the Kneedle approach with predicted value in logistic regression of lowest mean arterial pressure for postoperative delirium (POD). Figure showing the potential threshold prediction.
(TIF)

S1 File Checklist. This article adheres to the CONSORT checklist.
(DOC)

S2 File Original data. We have provided the original data of the study.
(XLSX)
