# Supplementary figures and images for: Association between intraoperative hypotension during brain tumor resection and postoperative delirium: A secondary analysis of a randomized controlled trial
Source: PLoS One. 2025 Oct 29;20(10):e0334094. doi: 10.1371/journal.pone.0334094 (PMC12571290; doi:10.1371/journal.pone.0334094)

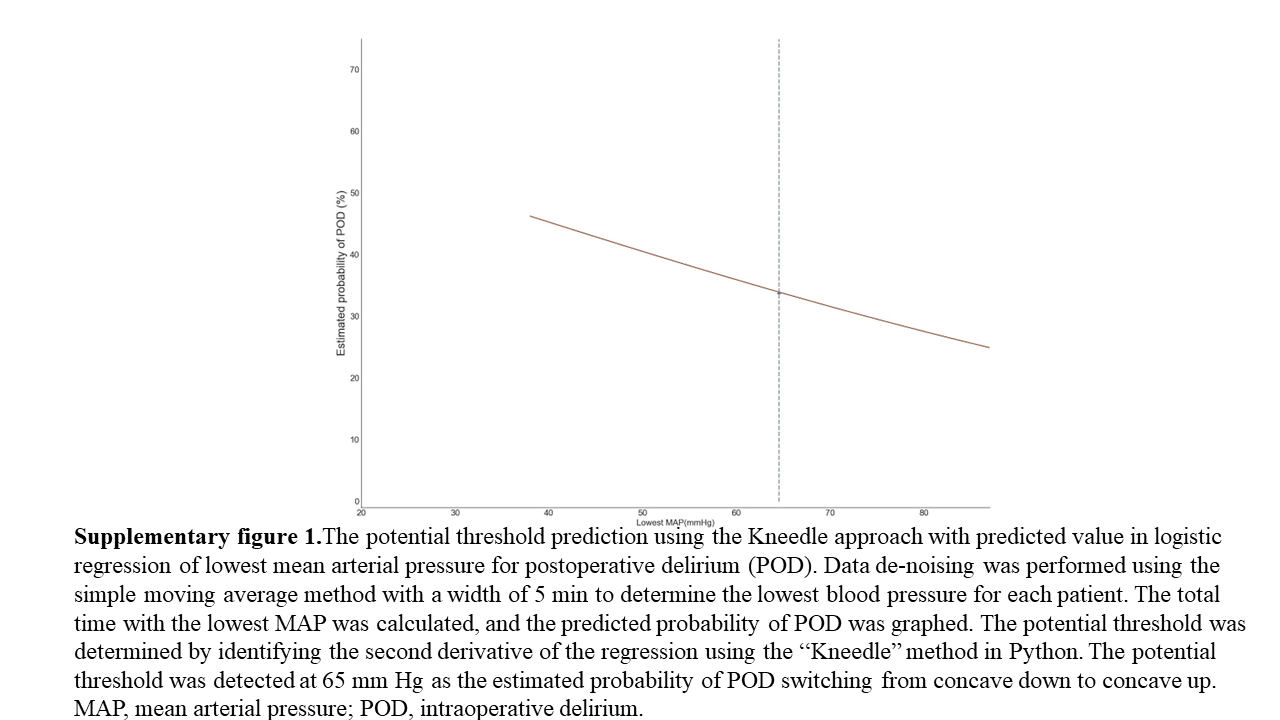

Supplement: S1 Fig — Figure showing the potential threshold prediction. (TIF) [file pone.0334094.s001.tif]
